# The Effect of COVID-19 and COVID-19 Vaccination on Assisted Human Reproduction Outcomes: A Systematic Review and Meta-Analysis

**DOI:** 10.3390/diseases12090201

**Published:** 2024-09-03

**Authors:** Andrea Milostić-Srb, Nika Srb, Jasminka Talapko, Tomislav Meštrović, Tihomil Žiger, Stana Pačarić, Rajko Fureš, Vedrana Makarović, Ivana Škrlec

**Affiliations:** 1 Faculty of Dental Medicine and Health, Josip Juraj Strossmayer University of Osijek, 31000 Osijek, Croatia; 2 University Centre Varaždin, University North, 42000 Varaždin, Croatia; tmestrovic@unin.hr; 3 Institute for Health Metrics and Evaluation, University of Washington, Seattle, WA 98195, USA; 4 Department for Health Metrics Sciences, School of Medicine, University of Washington, Seattle, WA 98195, USA; 5 Department of Surgery, University Hospital Centre Osijek, 31000 Osijek, Croatia; 6 Department of Gynecology and Obstetrics, Zabok General Hospital and Croatian Veterans Hospital, 49210 Zabok, Croatia; 7 University Hospital Center Osijek, 31000 Osijek, Croatia

**Keywords:** SARS-CoV-2, COVID-19, COVID-19 vaccine, ART, IVF, ART outcomes

## Abstract

The most discussed infectious disease is coronavirus disease 2019 (COVID-19), caused by the severe acute respiratory syndrome coronavirus 2 (SARS-CoV-2) virus. Many research endeavors have focused on the effects of the virus on reproductive organs, as these have also been shown to carry the receptors to which the virus attaches. The results of assisted reproductive technology (ART) have been significantly affected by the pandemic, with some in vitro fertilization (IVF) centers being closed due to the risk of further spread of the disease. According to World Health Organization statistics, 17.5% of adults worldwide suffered from fertility problems in 2023; in other words, one in six people in the world have reproductive health problems. As infertility is a growing problem in the modern world and new developments in assisted reproduction are always a topic of profound interest, it is important to understand the impact of SARS-CoV-2 on reproductive health. This systematic review aimed to examine studies describing patients undergoing ART procedures with a COVID-19-positive history and to shed light on the recent evidence on the safety of COVID-19 vaccination in the ART context. A meta-analysis was conducted to confirm the results of the systematic review. The results showed a significant difference in clinical pregnancy rates between the vaccinated and unvaccinated groups and an increased miscarriage rate in those with a COVID-19-positive history. However, no significant difference in clinical pregnancy and birth rates was found in participants with a previous COVID-19 infection. The results show that further studies and research are needed, even though the spread and impact of the virus have decreased. Evidence-based information for individuals and couples undergoing infertility treatment is vital to enable informed decision-making.

## 1. Introduction

Coronaviruses are highly contagious viruses that cause mild to severe respiratory infections [1]. The global pandemic caused by the severe acute respiratory syndrome coronavirus 2 (SARS-CoV-2) was declared by the World Health Organization (WHO) on 11 March 2020 [2]. SARS-CoV-2 uses angiotensin-converting enzyme 2 (ACE2) as a cellular receptor for viral entry, as well as a cellular protease called transmembrane protease serine-2 (TMPRSS2) [2,3]. Replication of the virus takes place in the nasopharyngeal epithelium and spreads to the distal alveoli [2,4].

After the infection, the human body activates pattern recognition receptors (PRRs) that recognize foreign bodies called pathogen-associated molecular patterns (PAMPs) and danger-associated molecular patterns (DAMPs) [5]. The Toll-like receptor groups TLR3 and TLR7 recognize the virus and produce several type I interferons and proinflammatory cytokines [2,4]. The interferons bind to the surface receptors of the cell and initiate a signaling cascade via the JAK-STAT pathway, thereby activating transcriptional regulation of hundreds of interferon-regulated genes like oligoadenylate synthetase 1 (OAS1), OAS2, and OAS3 [6].

Extrapulmonary organs such as the heart, liver, kidney, colon, esophagus, brain, breasts, placenta, gallbladder, and testes have mRNA expressions of both ACE2 and TMPRSS2; this explains why SARS-CoV-2 may be able to affect these organs [7,8]. The virus induces the release of ACE2, causes cytokine storms, and leads to oxidative stress and increased body temperature. All this can become a severe threat to the reproductive health and fertility of the host [9]. Apart from the fertility aspect, it is important to know that respiratory viruses can lead to multiorgan failure through activation of the complement system and cytokine storms, dysregulation of the immune system response, and infiltration of the inflammatory cells [10].

The SARS-CoV-2 virus affects the reproductive organs via the ACE2 receptor. ACE2 is mainly found in men in spermatogonia, Sertoli, and Leydig cells [11,12]. In Leydig cells, the physiological function of ACE2 is to regulate testosterone production and modulate the conversion of angiotensin II to angiotensin I, thus balancing the interstitial fluid volume. During coronavirus disease 2019 (COVID-19), host ACE2 receptors are saturated by binding with the virus, which increases the availability of angiotensin II as it is not converted [13]. Infection can lead to inflammation, possibly affecting spermatogenesis and testosterone production. The high expression of ACE2 indicates that SARS-CoV-2 enters the testicular interstitium via the blood and causes hormonal dysregulation. In infected COVID-19 patients, serum luteinizing hormone (LH) was significantly elevated, and the ratio of testosterone to LH and the ratio of follicle-stimulating hormone (FSH) to LH were decreased considerably [14,15]. Apart from the effects on hormones, there may also be damage to the testicular tissue, causing orchitis or orchiepididymitis [11,16,17,18]. Autoimmune orchitis can result from a triggered immune response in the testes [19]. A study conducted on knockout mouse lines found that sperm have reduced sperm motility, resulting in a lower fertilization rate in vitro. Those data showed that TMPRSS12, which is predominantly expressed in the testis, impacts the regulation of sperm motility [20]. Recent studies have shown a difference between the sexes regarding contracting COVID-19 [21,22,23].

In women, ACE2 and TMPRSS2 are expressed together in the endometrium, ovarian cortex, and ovarian medulla, especially in oocytes of different stages, as well as in the membrane of the trophectoderm, in the hypoblast, in epiblast cells in blastocysts, and partially in granulosa cells [3,24,25]. The cellular ACE2 receptor and TMPRSS2 protease expression increased with oocyte maturation, but ACE2 was expressed at all stages of follicle maturation [12,26,27]. Thus, there is a link between infection and ovarian dysfunction [8]. Moreover, binding SARS-CoV-2 to the ACE2 receptor might affect ovulation, the menstrual cycle, and pregnancy. It appears that cytokine levels correlate with the severity of disease in pregnant women with the virus [28]. The virus disrupts the microenvironment of the follicle, potentially affecting reproductive outcomes and interfering with embryo implantation and pregnancy itself [8,12,29]. Studies also show that it is not only pregnancy but also birth outcome that is affected by the virus. For instance, the incidence of preterm births and cesarean deliveries were above the international averages [30,31]. Even older studies concluded that the SARS virus can cause higher rates of spontaneous miscarriages, premature births, and intrauterine growth disorders during pregnancy [32]. Side effects of the COVID-19 infection in infants include respiratory distress and fetal problems, low platelets with abnormal liver function, and, in some cases, even death [33,34]. However, most studies agree that males were more affected than females [35].

According to the WHO statistics, 17.5% of adults worldwide suffered from fertility problems in 2023 [36]. The present review summarizes the current impact of SARS-CoV-2 and COVID-19 on fertility and assisted human reproduction procedure outcomes. These include the clinical pregnancy rate, miscarriage rate, and birth rate. The basic genetic characteristics of COVID-19, its receptors, its impact on human fertility, and assisted reproduction since the pandemic’s beginning are discussed based on recently published evidence.

## 2. Materials and Methods

### 2.1. Literature Search

The systematic review was conducted based on the Preferred Reporting Items for Systematic Reviews and Meta-Analyses (PRISMA) statement, and the study protocol is registered in the PROSPERO database (CRD42024520979). The literature included English libraries such as PubMed, NIH, EMBASE, iCite COVID-19 portfolio, Cochrane Library, Google Scholar databases, and WHO and ESHRE statistics. Aside from this, the review was not limited to English and thoroughly examined literature available in other languages to gain insight into practices in central and south-eastern Europe. The search included articles from 1 January 2020 until 1 December 2023. The search keywords were: COVID-19; SARS-CoV-2; COVID-19 vaccine; IVF; ART; assisted reproduction; infertility; in vitro fertilization; intracytoplasmic sperm injection; ICSI; outcome; pregnancy; embryo; implantation; and birth; these were combined using Boolean operators AND, OR, and NOT.

### 2.2. Eligibility Criteria

The main focus of this review was on the outcomes of COVID-19 patients undergoing ART treatment. Therefore, the inclusion criteria for a study were the description of original data on COVID-19-positive or COVID-19 vaccinated patients and the impact on their fertility. The outcomes included miscarriages, clinical pregnancy, and birth. Clinical pregnancy data were defined as the presence of an intrauterine gestational sac detected by ultrasound examination and the detection of human chorionic gonadotropin in serum. The miscarriage rate was characterized as the proportion of pregnancies ending in miscarriage within the first three months expressed as a ratio to the number of clinical pregnancies. The birth rate was calculated as the number of births per clinical pregnancy. All articles that included cohort, case-control, and cross-sectional studies were included. Studies that did not provide original data on fertility and COVID-19 were excluded. If the article described the pregnancy outcomes of patients with a positive COVID-19 history but did not abstain from a type of assisted reproduction, these studies were not included. If a study described the effects of the pandemic on infertile couples and not the virus or the vaccine itself, it was also excluded.

### 2.3. Data Extraction

The articles, papers, and statistical data were independently assessed by the two authors (A.M.-S. and N.S.), and each study was included or excluded from the list according to the criteria for inclusion and exclusion. Complete access to all data was given to every author in this research. Discrepancies were resolved by discussion.

### 2.4. Study Quality

To assess the risk of bias, the quality of the included studies was rated using the Newcastle–Ottawa Scale (NOS) [37]. The scale is divided into three parts. The parts include the Selection, Comparability, and Exposure for case-control studies. In the categories of Selection and Exposure, the maximum is one star for each numbered item in a study. However, a maximum of two stars can be given in the category of Comparability. The total number of items is eight, and a maximum of nine stars can be awarded for a study. For cohort studies, the categories include the Selection, Comparability, and Outcome. The same star system is applied. Though there is no validated rating, most researchers agree that studies with 7–9 stars are considered high quality, and those rated with 0–4 are at a very high risk of bias. The studies included in this review are divided into two tables based on the study type. For the cohort-study group, the mean value of the studies was 7.27 since most of the studies had been awarded 7 or 8 stars (Supplement Table S1). In the case-control study table, the mean value of the quality was 7.5, with one study being awarded a maximum of 9 stars. In contrast, one study was given six stars, the lowest-rated included study (Supplement Table S2).

### 2.5. Statistical Analysis

Meta-analyses were performed using Jeffreys’s Amazing Statistics Program (JASP) software ver. 0.18.3.0 (by the University of Amsterdam). Due to insufficient data, many studies could not be included in the quantitative analysis. Thus, only eight studies were included on the history of the SARS-CoV-2 virus infection and seven studies on the administration of vaccines. The value of the I2 index and Q-test assessed statistical heterogeneity. I^2^ values > 75% and the *p* value < 0.001 were considered high heterogeneity; thus, random effects models were used. Potential publication bias was examined using the symmetry of the funnel plot and rank correlation test. A forest plot, with the principal outcome of the odds ratio (OR) and 95% confidence interval (95% CI), was used to display the results. *p* ≤ 0.05 was considered statistically significant. Only data about the clinical pregnancy rate were subjected to analysis.

## 3. Results

**Figure 1:**
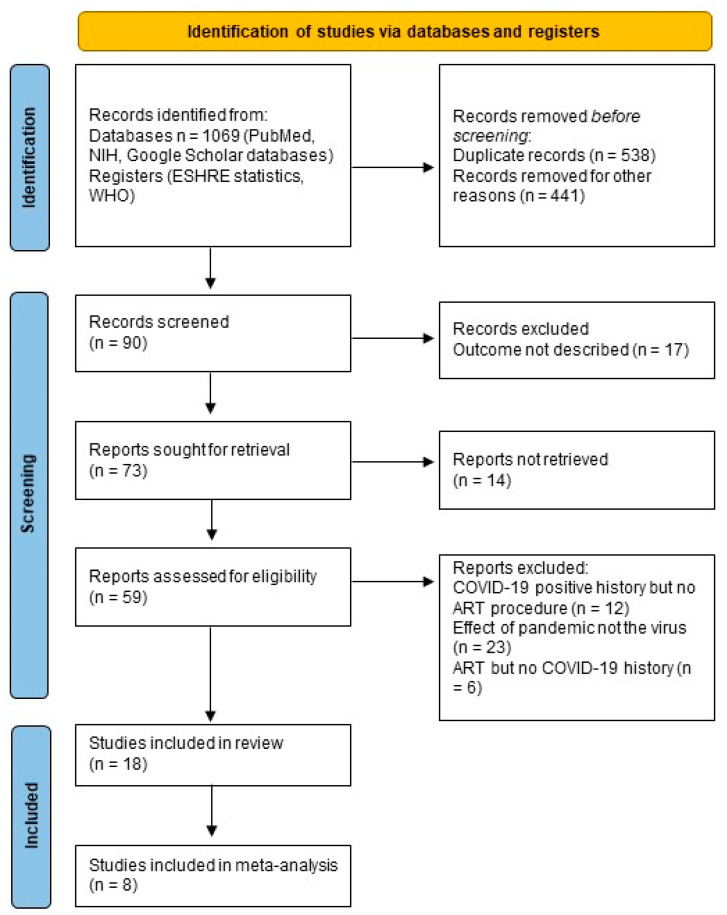
PRISMA flow diagram.

A total of 1069 articles were found in the databases. After removing duplicates and screening the titles and abstracts based on the eligibility criteria, our search identified 90 records. Articles describing the experience with ART treatments of COVID-19-positive patients but not providing data on treatment outcomes were also excluded. The relevant full-text articles were then reviewed and evaluated after the inclusion and exclusion criteria were applied, and 18 studies remained (Figure 1). Of these 18 studies, 11 described the effects of COVID-19 and 7 described the impact of the vaccine on ART outcomes of infertile couples. In addition, 15 studies were included in the quantitative analyses.

### 3.1. Characteristics of the Reviewed Studies

Although the total number of all participants cannot be added as some studies reported the number of ART treatment cycles instead of the number of participants, there were over 2096 participants with a positive history of COVID-19 or SARS-CoV-2 infection (Table 1). The exact number could not be calculated, as the study by Eckstein et al. [38] does not state the number of participants but rather the number of treatment cycles performed. The total number of vaccinated patients was 2362, with 4920 vaccinated couples (Table 2). The potential clinical outcomes of the treatments were miscarriage, clinical pregnancy, and birth. The included studies were published between early 2021 and October 2023. There were five observational, seven cohort, and six retrospective studies. Four were multicenter studies, and all reported at least one of the outcomes. Some even stated that specific data, such as birth rates, were unavailable during the study.

Approximately 562 pregnancies were recorded in the studies included in this review (Table 1). When calculating birth rates, the study by Banker et al. was not included because their data describe specific ART-like outcomes and not overall birth rates [48]. The same logic was applied when calculating the total birth rates in the studies. Thus, the number of live births is above 335. The same applies to the miscarriage rate, which is 188. In all studies, the participants were in their thirties according to their average age. This is consistent with other ART studies, which show that over 60% of ART patients are around 35 years old [49,50].

**Table 2:** Studies describing patients undergoing ART procedures vaccinated for COVID-19.

Study	Study Type	City, Country	Publication Date	No. COVID-19 Vaccinated Participants	Mean Age ± SD	Pregnancy	Birth	Miscarriage
Huang et al. [51]	retrospective cohort study	China	9 February 2022	66	33.7 ± 5.6	39/66 (59.1)		
Dong et al. [52]	prospective cohort study	China	27 June 2022	735 infertile couples	33.15 ± 3.55	70/132 (53.03)		
Wang et al. [53]	cohort study	China	16 December 2022	4185 couples	31.49 (3.70)	603 couples with clinical pregnancies		
Chen et al. [54]	retrospective study	China	11 January 2023	268 women with inactivated or recombinant COVID-19	33.32 ± 5.14 (inactivated) 33.00 ± 5.16 (recombinant)	(inactivated) 46/77 (57.94) (recombinant) 11/15 (3.33)	No data yet	(inactivated) 2/77 (2.60) (recombinant) 2/15 (13.33)
Yang et al. [55]	retrospective study	China	11 October 2023	899	30.71 ± 3.84	141		
Chillon et al. [56]	observational study	Austria, Germany	17 March 2023	45	35.53 (7.00%)	1 (2.2%)	6 (13%)	
Zhang et al. [57]	case-control study	China	8 August 2023	1084	32.00 (30.00–35.00)	248/1084 (22.88)	180/1084 (16.61)	70/504 (13.89)
TOTAL				2362 4920 couples		1159	186	74

SD—standard deviation.

The number of vaccinated patients undergoing ART in the studies included was 2362, and 4920 couples had received the vaccination. The average age of the participants in the included studies was similar and in line with the previously mentioned facts about ART patients [49,50]. As the studies were conducted relatively recently, only 186 births were recorded, of which 180 belonged to one study. The total number of pregnancies in the studies was 1159, and the number of miscarriages was 74. It is interesting to note that six of the seven studies were conducted in China.

### 3.2. Meta-Analysis

**Figure 2:**
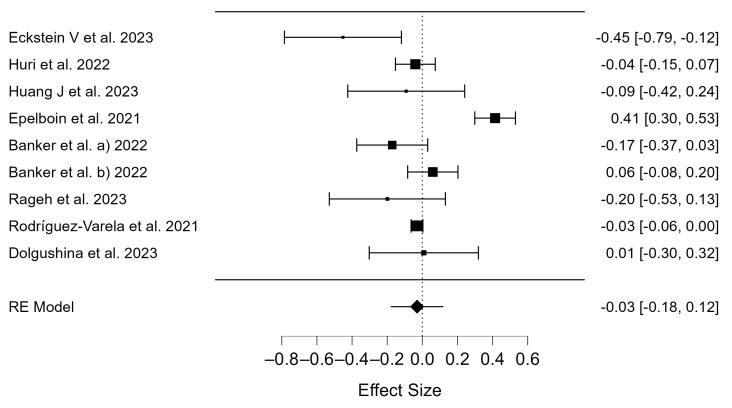
Forest plot of studies of COVID-19 positive vs. COVID-19 negative for clinical pregnancy [38,39,40,42,44,45,47,48].

A meta-analysis was conducted on eight studies of the COVID-19 history group, all of which had clearly described and presented their data. In the vaccine administration group, seven studies were included and quantitatively analyzed. The excluded studies did not provide information on the total number of pregnancies or birth rates, so the information was unavailable for our research. The data of 10,389 participants from the COVID-19 positive group and the 254,816 participants from the control group (the COVID-19 negative group) were pooled (Supplement Table S3). The Q-test and I^2^ index showed high heterogeneity in clinical pregnancy outcomes (*p* < 0.001, Q = 0.143; and τ^2^ = 0.043, I^2^ = 90.32). The high heterogeneity of the results becomes apparent in the forest plot (Figure 2). The random effects method was used as only a few studies were included. Thus, the differences in clinical pregnancy rates were compared for the COVID-19 positive and COVID-19 negative groups, and there were no differences (OR = −0.03; 95% CI: −0.18–0.12; *p* = 0.705; Figure 2). In Figure 2, study Banker et al. a) represents the fresh ET group and study Banker et al. b) represents the frozen ET group.

**Figure 3:**
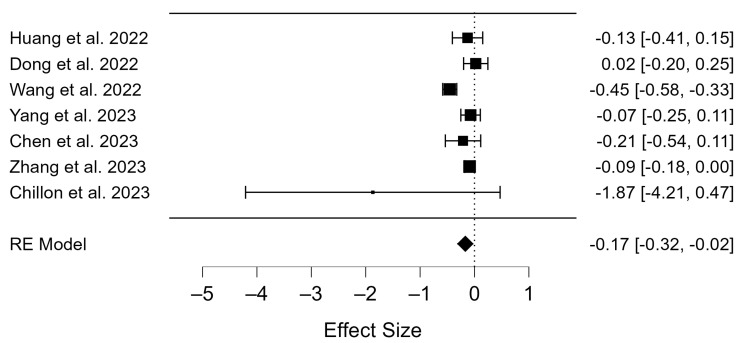
Forest plot of included studies on clinical pregnancy rate for vaccinated vs. not vaccinated participants [51,52,53,54,55,56,57].

The data from the vaccinated and unvaccinated groups were analyzed and the random effects method was used to test whether the COVID-19 vaccines affected clinical pregnancy outcomes (Supplement Table S4). The Q-test and I^2^ index showed substantial heterogeneity (*p* < 0.001; τ^2^ = 0.025, I^2^ = 74.04). This is visible in the graphical representation of the forest plot (Figure 3). Clinical pregnancy rates were compared between vaccinated and unvaccinated participants undergoing IVF. Statistically significant differences were found (OR = −0.17; 95% CI: −0.32–(−0.01); *p* = 0.029; Figure 3).

**Figure 4:**
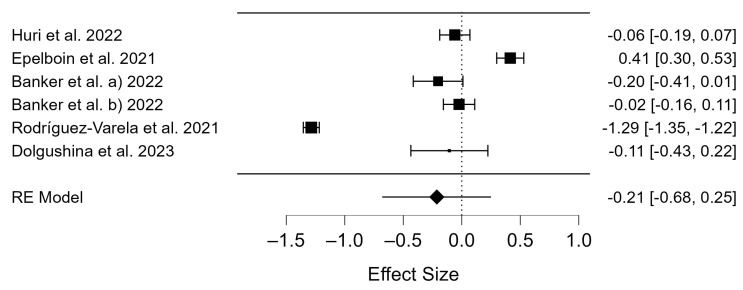
Forest plot of studies of COVID-19 positive vs. COVID-19 negative for birth rates [39,42,45,47,48].

Subsequently, the data of 10,096 participants were pooled for the birth rates of the COVID-19 positive group and 254,478 participants in the control groups (the COVID-19 negative group) (Supplement Table S3). As with pregnancy rates, the Q-test and I^2^ index showed high heterogeneity in birth outcomes (*p* < 0.001, Q = 0.809; and τ^2^ = 0.328, I^2^ = 98.69). The high heterogeneity of the results is displayed in the forest plot (Figure 4), and thus the random effects method was used. The differences in the birth rates were compared for the COVID-19 positive and COVID-19 negative groups and there were no differences (OR = −0.21; 95% CI: −0.68–0.25; *p* = 0.719; Figure 4).

**Figure 5:**
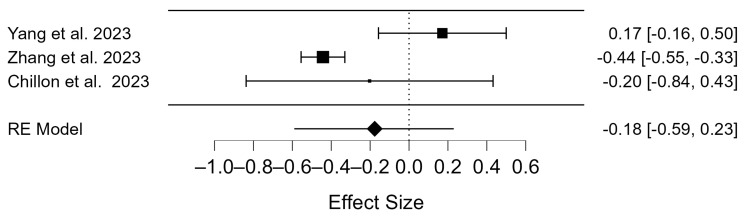
Forest plot of included studies on birth rate for vaccinated vs. not vaccinated participants [55,56,57].

The overall number of birth rate participants was 1292 in the vaccinated group and 1440 in the unvaccinated group. The data from the vaccinated and unvaccinated groups were analyzed using the random effects method to investigate whether COVID-19 vaccines affected birth outcomes (Supplement Table S4). The Q-test and I^2^ index showed considerable heterogeneity in birth rates (*p* = 0.002; τ^2^ = 0.097, I^2^ = 79.70). This is visible in the graphical representation of the forest plot (Figure 5). The birth rates of the vaccinated and unvaccinated participants were then compared. There were no statistically significant differences between vaccinated and unvaccinated participants (OR = −0.18; 95% CI: −0.59–0.23); *p* = 0.399; Figure 5).

**Figure 6:**
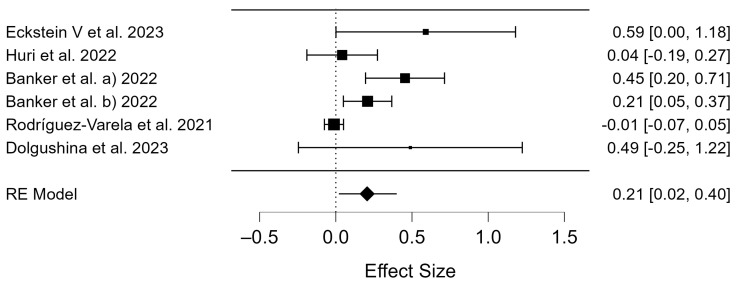
Forest plot of studies of COVID-19 positive vs. COVID-19 negative for miscarriage rates [38,39,42,47,48].

The number of participants in the miscarriage rates for the COVID-19 positive group was 4692 and 5754 for the COVID-19 negative group (Supplement Table S3). The Q-test and I^2^ index showed substantial heterogeneity in miscarriage outcomes (*p* < 0.001, Q = 4.514; and τ^2^ = 0.034, I^2^ = 77.49), which is displayed in the forest plot (Figure 6). The differences in the miscarriage rates were compared for the COVID-19 positive and COVID-19 negative groups, and there were significant differences (OR = 0.21; 95% CI: 0.02–0.40; *p* = 0.034; Figure 6).

**Figure 7:**
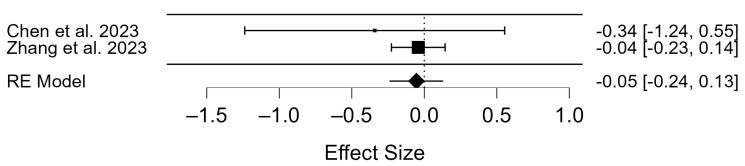
Forest plot of included studies on miscarriage rate for vaccinated vs. not vaccinated participants [54,57].

The total number of participants in the miscarriage rates for the vaccinated group was 581 and was 748 in the control group (the unvaccinated group) (Supplement Table S4). The Q-test and I^2^ index showed no heterogeneity in miscarriage rates (*p* = 0.520; τ^2^ = 0.00, I^2^ = 0.00). The birth rates of vaccinated and unvaccinated participants were compared. There were no statistically significant differences in birth rates between vaccinated and unvaccinated participants (OR = −0.05; 95% CI: −0.24–0.13); *p* = 0.561; Figure 7).

### 3.3. Publication Bias

**Figure 8:**
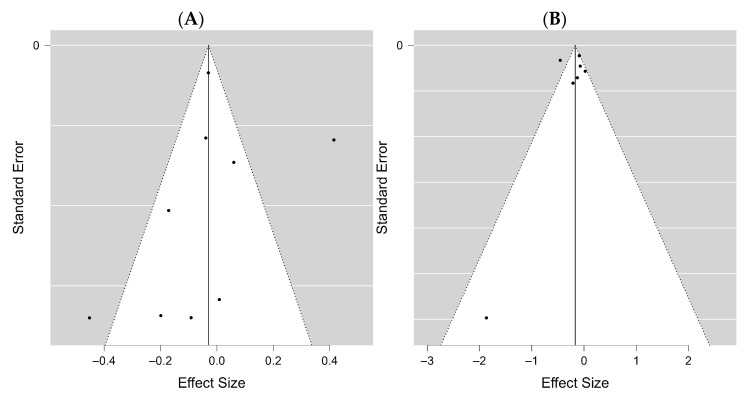
Funnel plots of clinical pregnancy rate. (**A**) COVID-19 positive vs. negative participants, (**B**) vaccinated vs. unvaccinated participants.

In addition, the potential publication bias was estimated for all included studies. The funnel plot of the studies included in the clinical pregnancy rate was approximately symmetrical in both analyses, COVID-19 positive vs. negative and vaccinated vs. unvaccinated, with Kendall’s τ values of −0.111 and −0.429 (*p* = 0.761 and *p* = 0.293), respectively (Figure 8A,B). Almost all studies are at the top in the vaccinated vs. unvaccinated groups, showing that these are studies with high strength.

**Figure 9:**
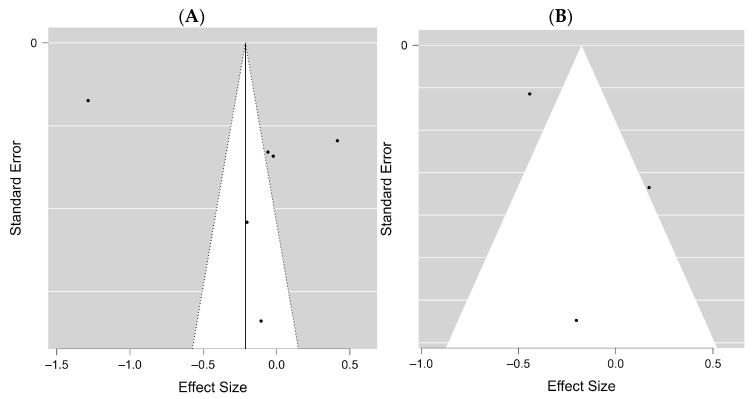
Funnel plots of birth rate. (**A**) COVID-19 positive vs. negative participants, (**B**) vaccinated vs. unvaccinated participants.

The funnel plot for the positive vs. negative birth rates of the COVID-19 groups is shown in Figure 9A and is mostly symmetrical with Kendall’s τ values of −0.200 (*p* = 0.719). Figure 9B shows the funnel plot for the vaccinated vs. unvaccinated birth rates, where Kendall’s τ values are 0.333 (*p* = 1.00), indicating no publication bias.

The funnel plot for the miscarriage rates of COVID-19 positive vs. negative groups is shown in supplementary Figure S1A. It is symmetrical with Kendall’s τ values 0.200 (*p* = 0.719). In supplementary Figure S1**,** the funnel plot for the miscarriage rates of vaccinated vs. unvaccinated groups is also displayed, with Kendall’s τ values of −1.00 (*p* = 1.00), indicating no publication bias.

## 4. Discussion

### 4.1. Clinical Pregnancy Rate of COVID-19 Patients

The studies examined over 500 pregnant women with a positive COVID-19 history (Table 1). The results of the meta-analysis showed no statistically significant difference in clinical pregnancy rates between the COVID-19 positive and COVID-19 negative groups (Figure 2). In 2018, the European Society of Human Reproduction and Embryology (ESHRE) announced that their results for that year showed a continued increase in the reported number of ART treatment cycles and children born in Europe [58]. In 2019, the year the pandemic began, ESHRE also reported an increase in ART treatment cycles and birth rates, with an increase in abandonment rates for fresh IVF or intracytoplasmic sperm injection (ICSI) cycles [59]. Frozen embryo transfers (FET) showed higher pregnancy rates (PRs), intrauterine insemination (IUI) cycles decreased with no change in outcomes, and there was a decrease in multiple embryo transfers [59]. In 2020, preliminary results were published, including 1157 IVF clinics in different countries and 1176 IUI units. These 35 countries reported that their clinical pregnancy rates (PR) per aspiration and transfer were similar to 2019 [60]. Data from the pandemic years have yet to be published. In China, where the pandemic began, 63.6% of ART treatment providers and 95.5% of sperm banks ceased operations due to the pandemic [61]. Wei et al. described data on clinical pregnancy and delivery rates, which showed a steady upward trend in the post-pandemic period [62].

### 4.2. Birth Rates of COVID-19 Patients

Over 300 births were described in the studies examined (Table 1). As previously mentioned, birth rates after ART procedures increased in the years before the pandemic [37,38,39,40]. In their systemic review and meta-analysis, Allotey et al. reported that COVID-19-infected pregnant women are more likely to experience preterm birth, have an increased risk of maternal death, and that babies are more likely to be admitted to the neonatal intensive care unit compared to women without a history of infection [63]. In 2018, it was reported that around 1.5 million IVF cycles are performed worldwide each year, resulting in around 400,000 live births, and that the overall rate of live births after ART is 0.3% [64]. A Canadian study found that the risk of COVID-19 for women of childbearing age appears to be low and that there is evidence of low risk for pregnant patients and their fetuses. In addition, the study suggested that ART procedures and fertility treatments should not have been postponed due to the pandemic [49]. Furthermore, our meta-analysis revealed no statistically significant differences in the birth rates of the two groups, namely those who tested positive for COVID-19 and those who tested negative (Figure 4).

### 4.3. Miscarriage Rates of COVID-19 Patients

Table 1 describes a total of 188 miscarriages in the considered studies. The results of the meta-analysis suggest that individuals infected with the SARS-CoV-2 virus may have an increased risk of early miscarriage during ART treatment (*p* = 0.034, Figure 6). These findings are consistent with those of a large cohort study from the UK, which showed that COVID-19 infection is associated with an increased incidence of first-trimester abortion and that pregnant women infected with the SARS-CoV-2 virus are at a higher risk of miscarriage [65]. An increased miscarriage rate in the ART procedure was observed in couples with a previous COVID-19 infection. Considering the high contagiousness of SARS-CoV-2, many questions have arisen regarding the risk of transmission to and between human embryos, gametes, and reproductive tissues during cryostorage in ART laboratories [49]. Opinions on the effects of COVID-19 on the embryo and fetus remain divided. Kotlyar et al. state in their study that vertical transmission of SARS-CoV-2 is possible and occurs in a few cases of maternal SARS-CoV-2 infection in the third trimester. The infection rates are similar to other pathogens that cause congenital infections [66]. On the other hand, Setti et al. reported that their data set showed no increased risk of miscarriage in the first trimester of ART pregnancies achieved during the onset of COVID-19 [67]. A Canadian review summarized the knowledge on the impact of SARS-CoV-2 infection on reproductive health [49]. No transmission cases during ART treatments, such as cryopreservation, embryo culture, or storage, were recorded, so authors have concluded that the likelihood of COVID-19 affecting embryos or their recipients is low [49].

### 4.4. Clinical Pregnancy Rate of COVID-19 Vaccinated Patients

The second table describes the vaccinated patients and their ART results. Approximately 1159 pregnancies were recorded in the studies examined. Opinions also differ on the safety of vaccines in terms of fertility. A study published in 2023 reported that pregnancy outcomes were not affected by taking the inactivated vaccine before frozen embryo transfer [57]. It also states that the number of vaccine doses and the interval between them had no effect, thus providing evidence for the safety of administering the vaccine during pregnancy in patients undergoing ART [57]. However, another reasonably recent study concluded that while the vaccine did not affect egg quality and embryo transfer, the impact on reproductive outcomes and health remains uncertain [68]. Nevertheless, Shi et al. have shown that vaccination before the IVF procedure is associated with a lower pregnancy rate [69]. Regardless of these results, the data from this meta-analysis showed a statistically significant difference between the vaccinated and unvaccinated groups (*p* = 0.029, Figure 3). It can, therefore, be postulated that the administration of the SARS-CoV-2 vaccine may have an impact on the frequency of pregnancies in individuals undergoing ART, with a significantly higher prevalence observed in those who had not received the vaccine.

### 4.5. Birth Rates of COVID-19 Vaccinated Patients

The total number of births in all included studies was 186. As divergent viewpoints exist regarding the vaccine’s effects on pregnancy, similar divisions emerge concerning its influence on overall birth rates among healthy individuals. When comparing the birth rates of vaccinated and unvaccinated participants undergoing IVF, no statistically significant differences were found in the present meta-analysis (Figure 5). According to Zhang et al., the vaccine protects the mother and child, reducing the likelihood of fetal infection after birth [57]. Another study comparing unvaccinated and vaccinated women undergoing frozen embryo transfer found that live birth rates did not differ between the two groups [70]. The study also showed that the length and weight of the newborns did not change after vaccination [70]. Another Chinese study, in which the subjects were divided into four groups according to the vaccination status of both partners, provided evidence that the vaccination had no negative impact on the live birth rate [15].

### 4.6. Miscarriage Rates of COVID-19 Vaccinated Patients

The overall rate of miscarriage in the studies was 74. A meta-analysis showed that the COVID-19 vaccine did not impact the risk of miscarriage, although the analysis was based on only two studies (Figure 7). Zauche et al. compared the natural miscarriage rate in the first six weeks of pregnancy and found that vaccination before or during pregnancy did not increase the miscarriage rate [71]. A 2022 study compared the pregnancy outcomes of unvaccinated and vaccinated patients undergoing ART and found no statistically significant difference in miscarriage rates between the groups [72]. As early as 2021, researchers showed that data from clinical trials indicate that the vaccination does not harm reproductive health or increase the number of miscarriages [73].

### 4.7. Study Limitations

The strict inclusion and exclusion criteria demonstrate the study’s validity, proven by the non-significant difference between the groups regarding the baseline characteristics. The main limitation of this study is the relatively small number of studies found, as the inclusion criteria were quite strict, and artificial human reproduction is still a newer branch of medicine that needs more research. Due to the strict inclusion criteria, there is a potential selection bias in the included studies. Additionally, the study’s timeframe is constrained by the relatively short duration of the pandemic. Likewise, the critical studies are limited, although the systematic review included a moderate number of participants. The variability in methodologies and definitions across the included studies may have also introduced heterogeneity, reflected in the synthesis of findings. The studies’ heterogeneity should be considered a limitation of the research. Furthermore, we only included clinical pregnancy rate data in the meta-analysis. Most included studies were conducted in Europe, but most studies investigating the effects of the COVID-19 vaccine on ART were conducted in China. Also, the number of couples undergoing ART in each study varied considerably, from 4000 to as few as 6, which can significantly affect the results of individual studies. In addition, many of the included studies did not consider potential confounding factors such as the presence of various comorbidities (diabetes, hypertension) and sleep disorders in couples undergoing ART. The included studies did not specify which type of SARS-CoV-2 virus was affecting their patients (e.g., Delta and Omicron), and so we cannot determine whether all viruses have the same impact on ART methods and what impact each form of the virus has on IVF outcomes. Another issue is that not all studies mentioned which vaccine type was administrated. Furthermore, some of the included studies did not provide data on the children’s births, as they were either published before birth or did not have these data. Therefore, an accurate assessment of the impact of COVID-19 and the vaccine in such studies is highly subjective. It is also worth noting that publication bias could have influenced the findings when studies assessing ART procedures in COVID-19 vaccinated patients are concerned; however, it was insignificant.

## 5. Conclusions

Overall, there is still much to discover about the effects of SARS-CoV-2 infection on reproductive organs. It is important to prioritize the health and safety of people with infertility issues. When examining those currently undergoing ART treatment, the infection and the vaccine are compelling topics that need to be explored. Further research is needed to investigate the link between infertility and COVID-19 thoroughly. The safety and potential effects of the vaccine are also not yet fully known, as the long-term consequences for reproductive health are not yet known. The couples and individuals in the studies analyzed were not initially affected by their coronavirus infection. Nevertheless, the results of this meta-analysis suggest that SARS-CoV-2 infection was associated with an increased miscarriage rate. At the same time, vaccine administration was associated with decreased clinical pregnancy rates. However, the limited number of studies included in the meta-analysis should be considered when interpreting the results. Providing accurate information to individuals and couples undergoing infertility treatment can help allay concerns and enable informed decision-making. Finally, fostering an ongoing dialog between healthcare providers, researchers, and patients can contribute to a better understanding of the evolving landscape of COVID-19 and its impact on reproductive health.

## Figures and Tables

**Table 1:** Studies describing patients undergoing ART procedures with a COVID-19-positive history.

Study	Study Type	City, Country	Publication Date	No. COVID-19 Positive Participants	Mean Age ± SD	Pregnancy	Birth	Miscarriage
Eckstein V et al. [38]	cohort-study	Germany	10 October 2023	1581 treatment cycles	35.8 ± 4.1 (23.1–49.5)	19/87 (21.84%)	not published yet	10/19 (52.63%)
Huri et al. [39]	retro-prospective cohort study	Florence, Italy	25 April 2022	749		219 (100%)	165 (22.02%)	98
Rageh et al. [40]	observational study	Manama, Bahrain; Kingdom of Saudi Arabia	12 September 2023	88	32.14 ± (4.773)	32 (36.4%)		
Engels Calvo et al. [41]	multicenter, prospective, observational study	78 Spanish centers	12 April 2021	74	39.6	74 (5.5%)		
Rodríguez-Varela et al. [42]	retrospective, multicentric, and double-arm study	Spain, Lisbon and Rome	21 December 2021	6	38.6	6		39 (4.5%)-overall miscarriage rate
ESHRE COVID-19 Working Group et al. [43]	retrospective, multicentric studies	32 countries worldwide	13 September 2021	105	33.7 ± 6.1	25	67	10 (12.5%)
Huang J et al. [44]	prospective cohort study	China	4 October 2023	252	32.3 ± 5.0	83 (70.3%)		
Epelboin et al. [45]	prospective clinical study	France	30 November 2021.	16	31.1 (±5.9)		16 (1.8%)	No data
Ziert et al. [46]	multicentric, prospective, observational study	Germany, Austria	19 April 2022	65	34.09 ± 5.12	65	57/65 (87,69%)	0/57 (0.0)
Dolgushina et al. [47]	observational prospective study	Russia	29 June 2023	135	34 (31–37)	39 (28.9%)	30 (22.2%)	31 (23.1%)
Banker et al. [48]	retrospective cohort study	Ahmedabad, India	30 June 2022	606	30.7	47.6% Fresh ET 68.7% Frozen ET	32.4% Fresh ET 46.3% Frozen ET	10% Fresh ET 6.73% Frozen ET
TOTAL				2096		562	335	188

SD—standard deviation; ET—embryo transfer.

## Supplementary Materials

The following supporting information can be downloaded at: https://www.mdpi.com/article/10.3390/diseases12090201/s1, PRISMA 2020 Checklist [74]; Table S1: Newcastle-Ottawa Scale (NOS)—for cohort study; Table S2: Newcastle-Ottawa Scale (NOS)—for case-control study; Table S3: Quantitative data based on the history of the SARS-CoV-2 virus infection of studies included in the meta-analysis; Table S4: Quantitative data based on the vaccine administration of studies included in the meta-analysis; Figure S1: Funnel plots of miscarriage rate. (A) COVID-19 positive vs. negative participants, (B) vaccinated vs. unvaccinated participants.
