# Supplementary material for: The Effect of COVID-19 and COVID-19 Vaccination on Assisted Human Reproduction Outcomes: A Systematic Review and Meta-Analysis
Source: Diseases. 2024 Sep 3;12(9):201. doi: 10.3390/diseases12090201 (PMC11431602; doi:10.3390/diseases12090201)
Supplement: Supplementary file 1 [file diseases-12-00201-s001.zip › Supplementary tables_ART COVID.pdf]

Supplementary tables

Table S1. Newcastle-Ottawa Scale (NOS) – for cohort study

Study	Item & score								
	**Representativeness of the exposed cohort (1)**	**Selection of the non-exposed cohort (1)**	**Ascertainment of exposure (1)**	**Demonstration that outcome of interest was not present at start of study (1)**	**Compare ability of cohorts on the basis of the design or analysis (2)**	**Assessment of outcome (1)**	**Was follow-up long enough for outcomes to occur (1)**	**Adequacy of follow up of cohorts (1)**	**TOTAL**
Eckstein V et al. [38]	✸	✸	✸		✸	✸	✸	✸	7 ✸
Huri et al. [39]	✸	✸	✸	✸	✸	✸	✸	✸	8 ✸
Engels Calvo et al. [41]	✸	✸	✸	✸	✸	✸	✸	✸	7 ✸
Huang J et al. [44]	✸	✸	✸	✸	✸	✸		✸	7 ✸
Epelboin et al. [45]	✸	✸	✸	✸	✸	✸	✸		7 ✸
Ziert et al. [46]	✸	✸	✸	✸	✸	✸	✸		7 ✸
Banker et al. [48]	✸	✸	✸	✸	✸	✸	✸		7 ✸
Huang et al. [51]	✸	✸	✸	✸	✸	✸	✸	✸	7 ✸
Dong et al. [52]	✸	✸	✸	✸	✸	✸	✸	✸	8 ✸
Wang et al. [53]	✸	✸	✸	✸	✸	✸	✸	✸	7 ✸
Yang et al. [55]	✸	✸	✸	✸	✸	✸	✸	✸	8 ✸

Table S2. Newcastle-Ottawa Scale (NOS) – for case-control study

Study	Item & score								
	**Is the case definition adequate? (1)**	**Representativenessof the cases (1)**	**Selection of Controls (1)**	**Definition of Controls (1)**	**Comparability of cases and controls on the basis of the design or analysis (2)**	**Ascertainmentof exposure (1)**	**Same method of ascertainment for cases and controls (1)**	**Non-Response rate (1)**	**TOTAL**
Rageh et al. [40]	✸	✸		✸	✸	✸	✸		6✸
Rodríguez-Varela et al. [42]	✸	✸		✸	✸✸	✸	✸	✸	8✸
ESHRE COVID-19 Working Group et al. [43]	✸	✸	✸	✸	✸	✸	✸	✸	8✸
Dolgushina et al. [47]	✸	✸		✸	✸	✸	✸	✸	7✸
Chen et al. [54]	✸	✸	✸	✸	✸✸	✸	✸	✸	9✸
Zhang et al. [57]	✸	✸	✸	✸	✸	✸	✸	✸	7✸

Table S3. Quantitative data based on the history of the SARS-CoV-2 virus infection of studies included in the meta-analysis.

Study	COVID-19 positive group			COVID-19 negative group			Pregnancy rate		Birth rate		Miscarriage	
	Pregnancy	Birth	Miscarriage	Pregnancy	Birth	Miscarriage	Cohen's d	SE	Cohen's d	SE	Cohen's d	SE
Eckstein V et al.	19/87 (21.84%) Y=19, N=68	not published yet	10/19 (52.63%) Y=10 N=9	59/152 (38.82%) Y=59, N=93	(19.8%)	16/58 (27.59%) Y=16 N=42	-0.4521	0.169966			0.5902	0.300674
Huri et al.	261/749 (34,85%) Y=261, N=488	165/749 (22%) Y=165 N=584	54/219 (24.7 %) Y=54 N=165	308/844 (36,49%) Y=308, N=536	202/844 (23.9%) Y=202 N=642	61/262 (23.2%) Y=61 N=201	-0.0396	0.057806	-0.0593	0.065886	0.0416	0.11818
Huang J et al.	83/118 (70.3%) Y=83, N=35			70/95 (73.7%) Y=70, N=25			-0.0916	0.169846				
Epelboin et al.		99/874 (11.3%) Y=99 N=775			13848/243771 (5.7%) Y=13848, N=229923		0.4145	0.059041	0.4145	0.059041		
Banker et al. a) Fresh ET:	81/171 (47.6%) Y=81, N=90	55/171 (32.4%) Y=55 N=116	42/171 (24.4%) Y=42 N=129	194/352 (55.1%) Y=194 N=158	143/352 (40.6%) Y=143 N=209	44/352 (12.4%) Y=44 N=308	-0.1713	0.103057	-0.2022	0.108291	0.4542	0.132245
b) Frozen ET:	299/435 (68.7%) Y=299, N=136	201/435 (46.3%) Y=201 N=234	114/435 (26.1%) Y=114 N=321	434/654 (66.4%) Y= 434 N=220	309/654 (47.2%) Y=309 N=345	128/654(19.5 %) Y=128 N=526	0.0597	0.073032	-0.0231	0.068381	0.2084	0.08103
Rageh et al.	32/88 (36.4%) Y=32 N=56			41/91 (45.1%) Y=41 N=50			-0.1991	0.168582				
Rodríguez-Varela et al.	3713/7732 (48.1%) Y=3713 N= 4019	3003/7732 (38.8%) Y=303 N=4729	668/3713 (18.0%) Y=668 N=3045	4323/8752 (49.4%) Y= 4323 N=4429	3480/8752 (39.8%) Y=3480 N=5272	792/4323 (18.3%) Y=792 N=3531	-0.0303	0.017217	-1.2859	0.03482	-0.0122	0.032011
Dolgushina et al.	39/135 (28.9%) Y=39 N= 96	30/135 (22.2 %) Y=30 N=105	9/ 135 (6.7%) Y=9 N=126	30/105 (28.6%) Y=30 N=75	27/105 (25.7%) Y=27 N=78	3/105 (2.9%) Y=3 N=102	0.0085	0.158572	-0.1058	0.167875	0.4892	0.374816

SE – standard error; Y – confirmed diagnosis of clinical pregnancy, live birth or miscarriage; N – the diagnosis of clinical pregnancy, live birth or miscarriage has not been confirmed

Table S4. Quantitative data based on the vaccine administration of studies included in the meta-analysis

**Study**	**Vaccinated group**			**Unvaccinated group**			**Pregnancy rate**		**Birth rate**		**Miscarriage rate**	
	**Pregnancy**	**Birth**	**Miscarriage**	**Pregnancy**	**Birth**	**Miscarriage**	**Cohen's d**	**SE**	**Cohen's d**	**SE**	**Cohen's d**	**SE**
Huang et al.	39/66 (59.1%) Y=39 N=27			712/1103 (64.6%) Y=712 N=391			-0.1277	0.142324				
Dong et al.	70/132 (53.03%) Y=70 N=62			170/327 (51.99%) Y= 170 N=157			0.0231	0.113882				
Wang et al.	191/603 (31.7%) Y=191 N=412			310/603 (51.4%) Y=310 N=293			-0.4549	0.065933				
Yang et al.	141/291 (48,45%) Y=141/ N=150	29/163 (17.8%) Y=29 N=134		150/291 (51,55%) Y=150 N=141	23/168 (13.7%) Y=23 N=145		-0.0719	0.091384	0.1713	0.167517		
Chen et al.	(inactivated) 46/77 (57.94%) Y=46 N=31		2/77 (2.60%) Y=2 N=75	87/127 (68.50%) Y=87 N=40		6/127 (4.72%) Y=6 N=121	-0.2108	0.16585			-0.342	0.457392
Zhang et al.	504/1084(46.49) Y=504 N=580	180/1084 (16.61%) Y=180 N=904	70/504 (13.89%) Y=70 N=434	621/1228(50.57) Y=621 N=607	347/1228 (28.26%) Y=347 N=781	92/621 (14.81%) Y=92 N=529	-0.09	0.046015	-0.4425	0.057359	-0.0415	0.094452
Chillon et al.	1/45 (2.2%) Y= 1 N=44	6/45 (13%) Y=6 N=39		0/44 (0%) Y=0 N=0	8/ 44 (18%) Y=8 N=36		-1.869	1.193871	-0.2027	0.323873		

SE – standard error; Y – confirmed diagnosis of clinical pregnancy, live birth or miscarriage; N – the diagnosis of clinical pregnancy, live birth or miscarriage has not been confirmed

A

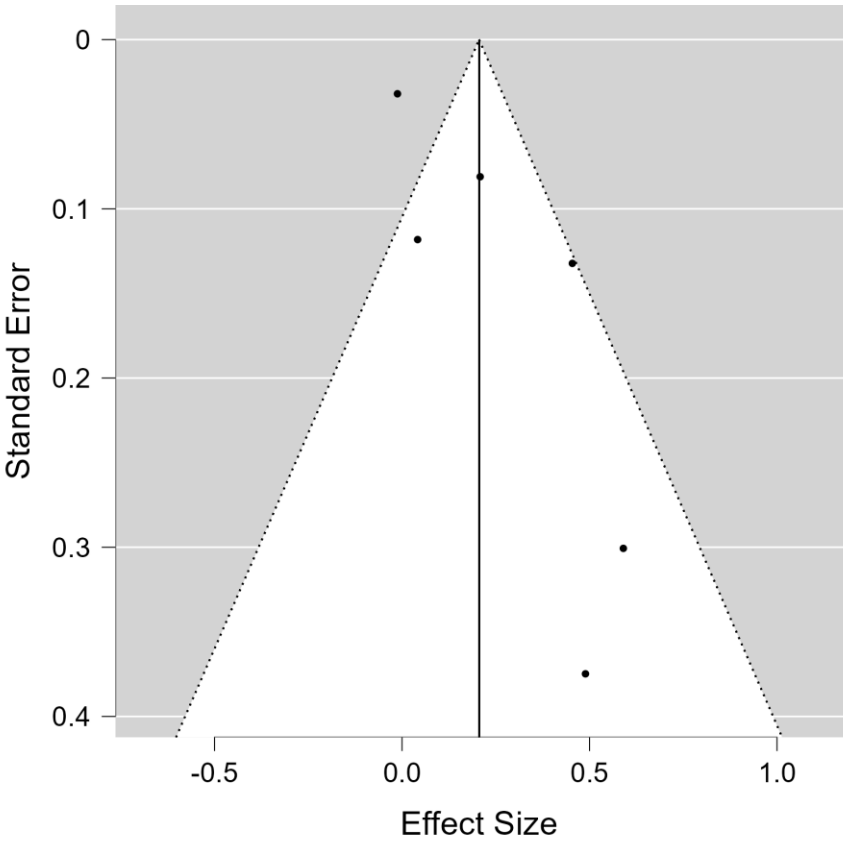

B

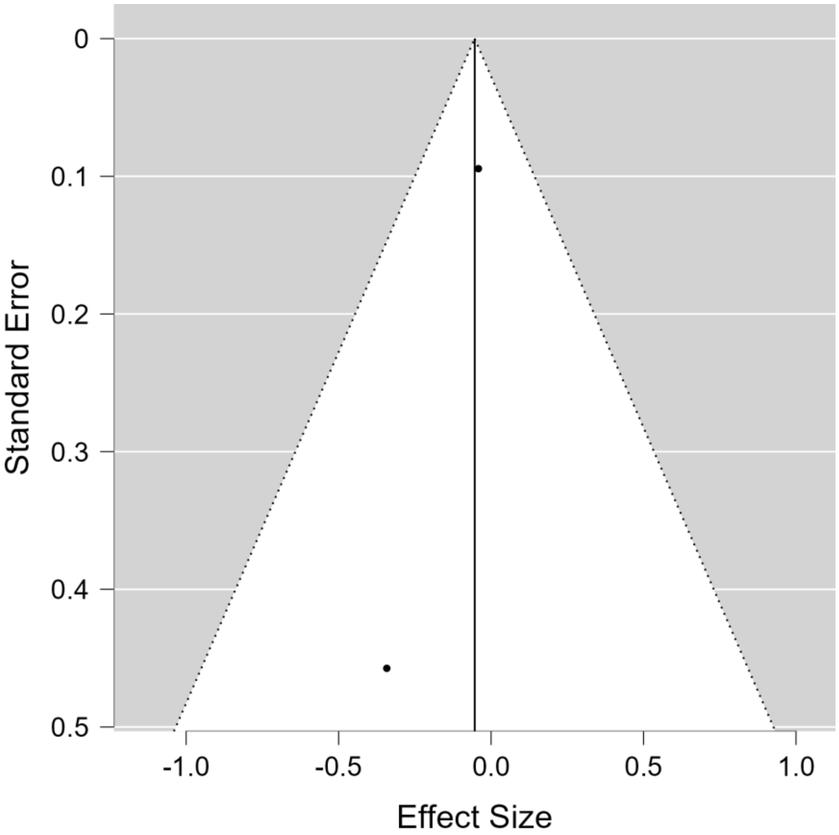

Figure S1. Funnel plots of miscarriage rate. A) COVID-19 positive vs. negative participants, B) vaccinated vs. unvaccinated participants.
